# Perceptions of clients on awareness and the geographical location of a South African university sexual health clinic

**DOI:** 10.4102/phcfm.v9i1.1350

**Published:** 2017-09-27

**Authors:** Rukshana Adams, Mariana M. Van Der Heever, Anneleen Damons

**Affiliations:** 1 Campus Health Service, Stellenbosch University, South Africa; 2 Division of Nursing, Stellenbosch University, South Africa

## Abstract

**Background:**

The Campus Health Service at Stellenbosch University has a sub-division, a sexual health clinic, which provides sexual health services. The clients of the sexual health clinic consist of staff members and students.

**Aim:**

This article reports on the perceptions of clients that relate to awareness and the geographical location of the clinic.

**Setting:**

The Campus Health Service at Stellenbosch University’s main campus.

**Method:**

A descriptive qualitative approach was applied utilising in-depth interviews. A sample of *n* = 15 was drawn through purposive sampling and data saturation was achieved with the sample.

**Results:**

The following themes emerged from the data: location of the clinic, awareness of sexual health services and marketing and advertising.

**Conclusion:**

The findings of the study revealed that accessibility of the clinic is influenced by the geographical location of the clinic and that marketing and awareness of services require attention.

## Introduction

Institutions of higher education can play a vital role in the promotion of sexual health. The World Association for Sexual Health recommends that health services, such as a university campus clinic, should provide convenient services that relate to sexual health needs.^1^

The findings of this article are part of a larger study that was conducted to explore the perceptions of clients on service delivery at the sexual health clinic. This article however reports on the findings of the larger study that relate to awareness and the geographical location of the sexual health clinic.

**FIGURE 1:**
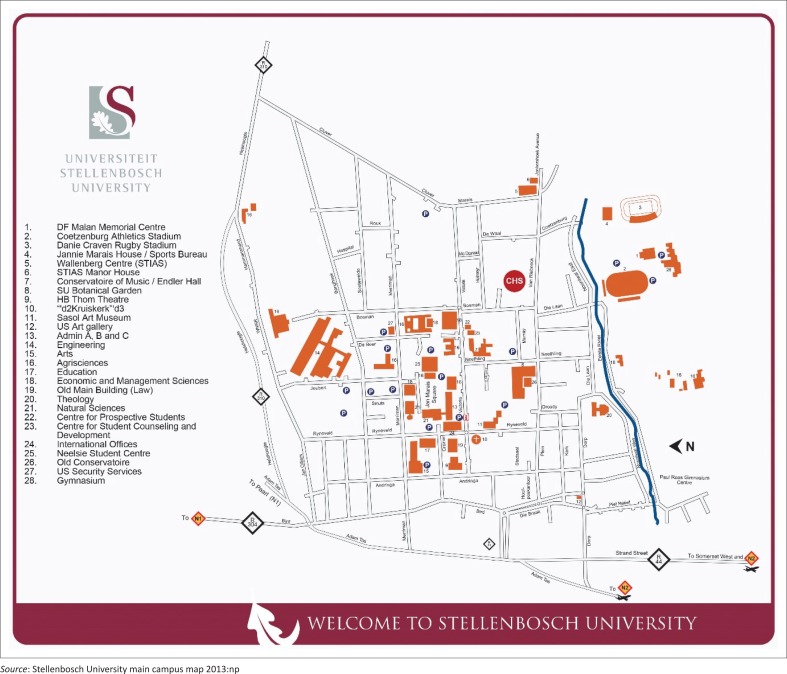
A map of the main campus, Stellenbosch University.^2^

The Campus Health Service (CHS) of Stellenbosch University (SU) is a health facility for students and staff and is situated on the university premises, yet off central campus. The location of the clinic is displayed in the accompanying map^2^ (Figure 1). The map illustrates that the central part of the campus is situated in the square formed by the four streets i.e.: Merriman, Andringa, Marais and Victoria streets. Most lecture halls, the library, student centre, administrative buildings and the language laboratory are situated in this central area. Sexual health services are provided by the sexual health clinic, a subdivision of services provided by SU CHS. CHS, thus the sexual health clinic, is situated in Claassen Street, on the other side of Victoria Street.^2^

Stellenbosch University has 31 university hostels, 2 private university affiliated hostels, as well as 8 independent private residences on campus.^3^ Students have access to the Stellenbosch public hospital which is situated 750 m from the main campus. Stellenbosch Medi-Clinic (a private hospital) is situated 3 km off the campus.^4^

Campus health services of other South African (SA) universities seem to be located in different areas of the university grounds. The Cape Peninsula University of Technology has six campuses; each campus has a clinic of which the campus health clinic is located in a central area such as close to the library or the administrative building.^5^ The campus health clinic of the University of the Witwatersrand in Johannesburg is situated in the building that hosts the university’s mall, close to student residences.^6^ Both the webpages of SU^7^ and the University of the Witwatersrand contain information regarding the type of services to be rendered and a map reflecting the location of the campus health clinic. The webpages do not contain specific information regarding sexual health. International universities such as Princeton University^8^ and the University of North Carolina^9^ also provide a map reflecting the location of the clinic, the variety of services that are available and costs pertaining to these services. In addition, the maps reflect accessibility in terms of easy access for wheelchairs and parking. The webpages of SA and international universities therefore assist in creating awareness of the location of the CHSs and the types of services that are available.

A survey was conducted to explore the views of students about service delivery at the CHS of Obafemi Awolowo University (OAU) Ile-Ife, one of the first-generation universities in Nigeria. The CHS of this university, OAU Ile-Ife, is located in the central campus area. The findings of the study showed satisfaction with the location of CHS at OAU Ile-Ife.^10^ The campus health clinic at Fitchburg State University in Massachusetts revised their services to be more accommodative for male students and relocated the facility to be close to student residences. The authors report that the new location in the proximity of the residences enhanced accessibility of the services.^11^

Accessibility of a service relates to the geographical location of the service and how convenient it is for clients to attend the service in terms of time and distance.^12^ The geographic context plays a critical role in health outcomes because it is associated with the utilisation of services. Increased distances to health care facilities negatively reflect poor accessibility of service.^13^ The factsheet of the World Health Organization on sexual health services advises that such services be conveniently located to enhance accessibility of target groups.^14^ The National Strategic Plan (NSP) of the South African Department of Health (2012–2016), which aims to address the challenges pertaining to sexual health, is tailored to ensure the rollout of structures to rural and urban areas.^15^

Kamau^16^ reports on factors that influence the utilisation of reproductive services in Kenya and avers that the lack of decentralised health facilities causes disparities in the provision of healthcare. A qualitative study completed in Sweden on youth-friendly services confirmed that sexual health clinics should be conveniently located to enhance the utilisation of services. For many young people the location of the service prevents them from attending the service. For example, some prefer that the clinic should be separately located from the central health facility. Yet, once the clinic is known to the community, some youths are also uncomfortable that they be recognised by people who know them.^17^

In addition, it is imperative that they have correct information regarding the location of the service, as they are embarrassed and uncomfortable to request directions to sexual health services. Hereafter, they are reluctant to seek professional healthcare assistance. Likewise, young people may delay seeking sexual health service when they have incorrect information regarding the location of a service or their eligibility for healthcare.^16^

Awareness of sexual health information and sexual health services influences the attendance of the service. The authors found in their study that the main source of sexual health information was through peers. The majority of participants revealed that they became aware of sexual health related information and services via close friends. The authors identified that one of the reasons for not attending services include unawareness of where to seek assistance.^18^

Currently marketing and advertising of the sexual health clinic at the university under discussion takes place via social media, such as Twitter and Facebook, presentations on screen in the waiting area and university intranet. Marketing tools such as pamphlets, billboards and magazines are beneficial strategies to promote sexual health services as proposed by Singh and Begum.^19^

### Problem statement

As mentioned in the rationale, the accessibility of sexual health services is influenced by the geographical location of a clinic, whether people know about the healthcare services rendered and how it is marketed. No previous studies were conducted that relate to the service rendered or factors influencing accessibility at the clinic. Therefore, substantial evidence that relates to the sexual health service and accessibility from the clients’ viewpoint was absent. Subsequently this article reports on the findings relating to awareness and geographical location.

### Aim

The objectives were to explore the perceptions of the clients attending the university sexual health clinic on the awareness of the sexual health clinic and knowledge of the geographical location.

## Research methods and design

The current study followed a descriptive design with a qualitative approach that allowed the researcher to identify and elaborate on the participants’ description of their perceptions of the sexual health clinic under study. The approach provided an opportunity to gain more information, understand and interpret their viewpoints and needs regarding awareness and the geographical location of the clinic.

The research study was undertaken on the main campus at SU CHS, South Africa. According to the 2013 SU CHS Statistics, the clientele attending the services at the main campus, during the period between March 2013 and September 2013, was large (*N* = 1146). Yet, this large total did not form the accessible population of the study. The first author is employed as a professional nurse at the clinic and provides sexual health services to the clients. The Protection of Personal Information Act^20^ however requires that personal information of clients be protected and not be used by employees who have access to it. The first author therefore approached participants who met the sample criteria after their sexual health consultation and requested permission from them that they be contacted for research purposes. The inclusion criteria relate to students and staff members who had accessed the sexual health clinic at the main campus more than once. These clients would potentially have broader perceptions of the clinic than a client who has accessed the clinic once or never before. Furthermore, the inclusion criteria incorporated students and staff members who had accessed the clinic for either STI treatment, HIV counselling and testing or for contraceptive methods. These categories were included as they constituted the range of services that are available at the sexual health clinic. Participants who initially consented to be contacted for research purposes, yet were not available at the time of data collection because of illness or class schedules were excluded from the study. The researcher confirmed with the prospective participants that they indeed meet the inclusion criteria before continuing the process. The potential participants were requested to complete a form in which they consented to be contacted for research purposes. The permission form to be contacted was then completed by the participants after a short introduction to the study. This document was not the informed consent document to participate in the study, but merely permission that they be contacted for research purposes.

A total of 21 clients who attended the sexual health services consented to be contacted. The 21 clients constituted the total accessible population. Purposive sampling was used to select 15 key participants. It allowed the researcher to select the sample based on the knowledge that each individual participant has of the phenomena under study.^21^

The first five participants’ viewpoints varied. Information obtained were of a general nature and not distinct. It was evident that the participants who are staff members had resembling perceptions regarding the phenomena of the study and limited in-depth information was obtained. Yet, information obtained from participants who were students differed. Consequently, the researcher recruited more participants in order to retrieve a more descriptive elaboration regarding their range of experiences. After the tenth interview, it was clear that information began to follow a distinct trend.

### Instrumentation

Data collection was completed by means of in depth interviews and a semi-structured interview guide which was based on the objectives of the study. The guide focuses on the perceptions of the clients that attended the sexual health services. The interview guide was not used in previous studies and was merely based on the objective of the current study, meaning the awareness of sexual health services and the geographical location of the clinic. It also enabled the participants to provide a fuller picture about the phenomenon under study.^21^

A pilot interview was conducted with one participant who met the inclusion criteria. The pilot interview uncovered no difficulties. The interview gave the researcher an opportunity to anticipate the length of time of each interview. The questions were clear and the participant understood what was expected. The fieldworker utilised the technique of reflection. The technique of reflection concerns Rogerian principles, a client-centred approach, in which the interviewer displayed unconditional positive regard towards the participant.^22^ In addition, the fieldworker summarised the information received and reflected it back to the participant. Information that was received, yet not clear, was followed up by means of probes. In these cases, the fieldworker asked that the interviewee elaborate on these unclear issues.^21^

### Trustworthiness

The truthfulness of the findings was enhanced by the criteria of credibility, transferability, dependability and conformability.^21^ Transferability relates to the extent to which the interpretative account can be applied to other contexts than the one being researched.^23^ The possibility that the findings be transferable to other settings can be enhanced by providing detailed information on the research procedure that was used.^24^ Therefore, as far as possible, detailed descriptions and a thick database are provided on the research process, for example population and sampling as well as data collection and analysis. Dependability is the degree to which the findings of a study are repeatable.^23^ An assessment was performed by an enquiry auditor to determine how well the research was conducted and whether the research process was logical, well documented and audited.^24^ Dependability is achieved through robust descriptions to indicate how certain actions and opinions are rooted in and developed out of contextual interaction.^23^ Therefore, the methods used in the study were provided with rich and detailed descriptions to convince others that the findings did indeed occur as related by the researcher. The findings of the study were clarified and verified by the researcher and fieldworker. Conformability is the degree to which the findings can be confirmed by others.^24^ Therefore, the interviews were transcribed verbatim. The resembling themes were reviewed by the supervisor of the study and where divergence existed, the researcher and supervisor reviewed the transcripts until concordance was reached.

### Data collection

Data collection commenced in August 2013 and was completed early September 2013. As the researcher is employed at the sexual health clinic as a professional nurse, in order to prevent bias, a fieldworker not affiliated to the university conducted the individual interviews. The fieldworker received training on the techniques and principles of interviewing, attended communication skills courses and assisted in previous qualitative interviews. In addition, the fieldworker received training on the conduction of interviews utilising Rogerian principles.

A short introduction of what the research study entailed was verbally presented to the participants again to ensure that they understood what the study entails. Voluntary consent to participate in the research study was completed by each participant during this consultation. Fifteen interviews were conducted at SU CHS boardroom. A tape recorder was used to audiotape all interviews.

Confidentiality was secured by protecting all data gathered within the scope of the project from being divulged or made available to any other unauthorised person.^21^ Only the researcher and supervisors involved had access to the data. Participants were assured that information obtained would not identify them personally. Therefore, participants were not referred to by name during the interviews. In addition, the transcribed interviews were nameless and labelled according to codes, for example interview 1, interview 2. Furthermore, there were no risks involved to participants’ confidentiality and anonymity as the interviews were personally transcribed by the researcher. All written notes and transcripts of the interviews are kept in a locked safe for at least 5 years.

### Data analysis

The interviews were transcribed by the researcher within 24 h of each interview. Field notes were typed and organised, which enhanced the researcher’s immersion in the data.^24^ The interpretive approach and the principle of bracketing described by Terre Blanche et al. was utilised during the data analysis process.^23^ The principle of bracketing was applied to ensure that the researchers’ personal concepts and beliefs regarding awareness of services at SU CHS did not influence the results of the study. All personal preconceived experiences and opinions were set aside with the intention of engaging in the new information obtained from the participants. In order to achieve familiarisation and immersion, the transcripts were read repeatedly and the recordings listened to repeatedly. Thereafter, the researcher identified and conceptualised phrases that represent the phenomenon as well as the induction of themes. These concepts were grouped and named according to similarity that represents the research topic. While the researcher remained focused on the intention of the study, main themes and subthemes were generated and labelled. Different sections of the data were marked as being instances of, or relevant to, one or more of the themes.^23^ Continuous coding and elaboration took place until no new insights emerged. Interpretations were put together in a written account of the phenomenon being studied using thematic categories.^23^ Therefore, the interpretations were reviewed by the fieldworker, supervisor and co-supervisor of the study. Finally, saturation was met as no new themes emerged from the data and it was decided that the information received is indeed sufficient, as it enabled the researcher to answer the research question.

### Ethical considerations

This article is based on an original research project approved by the Health Research Ethical Committee, Stellenbosch University, South Africa (ethical clearance number: No. 09/09/254). The study was not supported by any external funding.

## Results

Demographic details: Students – A total of 10 participants were students of which nine were female and one was male. The age of the students ranged between 20 and 25 years. Staff – A total of five participants were staff members all of whom were female. The age of staff members ranged between 25 and 45 years.

The total number of participants included in the study was 15 (*N* = 15) and consisted of one (*n* = 1) man and 14 (*n* = 14) women. Upon recruitment of participants, men were more reluctant than women to participate or talk about sexual health issues. The majority of the clientele who access the sexual health clinic are female. Subsequently, women have developed a sense of comfort more than men with regards to openness, because of positive building of relationships with staff members. Therefore, female responses compared to the male participant were more in depth. In addition, the female participants could elaborate more on the sexual health clinic because of the fact that the services included their monthly contraceptive methods.

Themes and sub-themes: Various sub-themes emerged from the major themes (see Table 1). The first theme relates to the location of the clinic. The second theme, awareness of the sexual health clinic, comprised four sub-themes which include unawareness of the sexual health clinic, informed of services by relative/peer or other informants, staff members, services that are available free of charge. The third theme, namely marketing and advertising of the service, included sub-themes such as insufficient marketing and CHS staff involvement in first-year student orientation.

**TABLE 1:** Themes and sub-themes.

Themes	Sub-themes
Location	Geographical distance/location
Awareness of the sexual health clinic	Unaware of sexual health clinic Informed by relative/peer/other Staff members Services available free of charge
Marketing and advertising of the service	Insufficient marketing Involve campus health service staff in first-year student orientation

### Location

Stellenbosch resembles a university town with residences and lecture halls that are situated over a wide geographical area. The size of the SU main campus grounds is 4 481 667 m^2^, which includes all areas owned by the university (Willoughby M 2014, personal communication, January 23). The CHS is located in Claassen Street, in the vicinity of the student residences that are located off the central campus towards the eastern vicinity of Stellenbosch. Yet, a variety of student residences are situated on the north, south and west side of the town. Only one of the entrances to Claassen Street has a direction board stating that CHS is situated in this road. The direction board however does not specify the range of services that are available at CHS. There are no direction boards from central campus to the clinic to guide the campus community to the clinic. Students residing in residences that are not in the vicinity of the CHS have to walk long distances to the clinic:

‘I’m currently staying in Goldfields residents, it’s like 20 minutes’ walk, it’s quite far … it’s, it’s difficult to get here because I have to walk a long distance.’ (Participant 2, female student)

‘There was a problem the first time, I was like where is this place.’ (Participant 1, female student)

‘…A smaller clinic that’s accessible, maybe in the Neelsie [student centre] that’s a bit more central.’ (Participant 15, female student)

‘…This is on campus but it’s off central campus like you have to make it visible on that side, not on this side, cause only people who live here come on this side.’ (Participant 11, female student)

‘I think they should … I don’t know … think they can move to any place closer to campus.’ (Participant 2, female student)

‘… She didn’t have any clue of where it is.’ (Participant 8, female student)

The CHS are not centrally located and directions to CHS from the main lecture halls, the administrative buildings of the university, and the residences on the opposite side of the main lecture halls, are currently absent. Subsequently a student who lives off campus or not in the residences close to the clinic might not know where the clinic is located. The possibility of visiting the clinic during lunch break depends on the location of their classes. The lecture halls are situated over a wide geographical area and some are not within walking distance of the clinic. The current geographical location of the clinic could therefore influence accessibility of services rendered at CHS.

### Awareness of the sexual health clinic

#### Unaware of sexual health clinic

Irrespective the location of CHS, accessibility is further influenced by the extent of awareness about the presence of a sexual health clinic. It became evident that various participants, students and staff members, were not knowledgeable of the sexual health clinic that is available at the CHS:

‘I mean … I’ve always been aware that the … campus health service is here … but not the sexual health clinic.’ (Participant 3, female staff)

‘She sort of didn’t hear about it (clinic) before either.’ (Participant 8, female student)

‘…The first-year students don’t know … because I told some of them today that I’m on my way to CHS, and they asked … CHS? Where is that? They didn’t know where the CHS is.’ (Participant 13, male student)

‘This is my 9th year, I think I’ve worked 7 years here before I found out about the clinic.’ (Participant 4, female staff)

Student and staff participants had varied perceptions regarding awareness of the clinic. Some participants knew about the campus health clinic, however were not aware of the sexual health clinic that is available. Yet, some claimed that they were completely unaware of the campus health clinic as well.

#### Informed by relative, peer or other

Insufficient knowledge about the specific sexual health services offered was reported by all the clients. Clients concluded that they became aware of the specific services offered by word of mouth and upon consultation with staff members employed at CHS:

‘I was only aware of that because I, think I was speaking to one of the doctors and they said well do you know we offer pap smears here as well. I mean I wasn’t even aware that that service was available.’ (Participant 3, female staff)

‘You don’t really know until you come here or you find out.’ (Participant 11, female student)

‘Also I’ve never actually came here for an HIV test because I’m also not sure do you just go and ask to see a nurse or what’s the procedure.’ (Participant 6, female student)

‘The doctor, she asked me when was the last time you had a pap smear, so I told her at my gyne that day. Then she said you’re not gonna do it anymore, from now on, all your sexual related problems, you come to the clinic here.’ (Participant 4, female staff)

In addition, the students who live in the vicinity of the CHS, report that they became aware of the service merely because they have to pass the clinic on their way to class.

‘I lived in a res down the road, so I always use to pass here.’ (Participant 6, female student)

It became clear that the location of the clinic in relation to the location of where participants lived on campus influenced their awareness of the service.

#### Staff members

In 2009, a formal launch of the CHS was presented to introduce the services to staff members. Prior to 2009, services were reserved for students only. Yet, participants who were staff members were not aware that the services are available to them.

‘…Because the people isn’t aware that the permanent, employed employees at Stellenbosch University is allowed to use this facility. They think it’s only for the students.’ (Participant 4, female staff)

‘And she’s working here more than 10 years [manager] for the university … she also didn’t know that there’s a service for, for us.’ (Participant 4, female staff)

‘I’m not really aware, I fell in to this because, I was here to see the other doctors. So, I wasn’t really aware of the services they offered here, e.g. the pap smear which you know I’m sure a lot of female students as well, could and should be making use of.’ (Participant 3, female staff)

The usage of services at the clinic is thereby seemingly related to the extent of awareness among potential clients. It is evident that various participants were unaware regarding specific services, especially sexual health services that are rendered at the clinic. The lack of awareness among staff members suggests a need for ongoing informative efforts.

#### Certain services are free of charge

The lack of awareness is also evident with regard to certain contraceptives and medication that are subsidised by the Department of Health in South Africa. These medications are available free of charge at the sexual health clinic. Various participants were not aware that certain services such as particular contraceptives, HIV testing and antibiotics are available free of charge at the clinic:

‘So I feel like people do need to know what they offer and they do need to make it a bit like, they just have to let you know that you can come for … let you know what, I feel like is, like an AIDS test is free. Um like cert … if you certain antibiotics like for STDs and stuff they are free. I feel like people knew that, they would come here.’ (Participant 11, female student)

‘I think it should be done in other ways, especially on res maybe because at the beginning you don’t know that if you come see a sister then you don’t pay, but if you see a doctor you have to pay, you have to pay cash, so the details you don’t know.’ (Participant 6, female student)

Participants claimed that when they initially enrolled at the university, they were not informed about the services during the orientation period. However, other participants claim that they had been orientated and informed during the orientation period and even physically brought to CHS by their mentors. It therefore seems that not all mentors involved in the orientation of students are thorough, or perhaps knowledgeable, about the availability of the service.

### Marketing and advertising of the service

#### Insufficient marketing

The participants revealed that a lack of knowledge existed regarding the availability of services at the CHS. The lack of knowledge is seemingly related to insufficient marketing and advertising strategies of the services:

‘I think they should do more campaigns around university, they already do the campaigns but, you know it’s not enough, like maybe once a year they do like testing. But then they, they should actually do like presentations around faculties or they should do campaigns and so that people can know about it.’ (Participant 2, female student)

‘I haven’t seen posters of the place.’ (Participant 9, female student)

‘Like he always came here for a doctor and he didn’t even know that you get the nurse option. Like certain things I do feel like … You don’t really know until you come here or you find out, cause … and he’s also on his 3rd year and he never knew that up until last that you can actually come here to see a nurse.’ (Participant 11, female student)

Irrespective of possible insufficient marketing some participants claim that when they initially enrolled at the university, they were not informed about the services during the orientation period. However, other participants claim that they had been orientated and informed during the orientation period and even physically brought to CHS by their mentors:

‘The location was not hard to find because she was showed physically by mentors during orientation week.’ (Participant 2, female student)

#### Involvement of CHS staff during first-year student orientation

First-year students are informed about the CHS during orientation week at the beginning of each academic year. During the orientation week, senior students have a treasure hunt day, of which one of the treasures to find is the CHS:

‘So, but she didn’t have any clue of where it is and she sort of didn’t hear about it before either. So maybe they could include that in the welcoming program for the first years. That could potentially, like I don’t know, sort of introduce them into the whole idea that there is doctors on campus.’ (Participant 8, female student)

It appears that marketing and advertising play a major role amongst participants as a means of being informed about campus issues. Likewise, an introduction about the CHS might be more efficient when staff members from CHS represent the clinic during orientation. According to participants, marketing and advertising would greatly benefit clients who live off campus and would be particularly informative specifically for the first-year students.

## Discussion

Various staff members and students were not aware of the existence of the CHS, the variety of services that are available, specifically the sexual health clinic and the availability of services that are free of charge. Awareness is seemingly created by word of mouth. Various participants had problems locating the clinic because the clinic building is not centrally located. Furthermore, there are no direction boards from the main campus to the clinic. Students and staff members were seemingly unaware regarding the specific eligibility criteria to access the sexual health clinic and some were completely unaware of the sexual health clinic and the service that is offered. Though various participants have accessed the CHS for different ailments, they were unaware that a sexual health service inside the same clinic facility is available.

Television, newspapers, internet, billboards, radio, family and friends are the main modes for students to obtain health related information.^19^ Therefore, modes of communication such as the campus magazine, the university intranet and billboards in the vicinity of the university could assist in creating awareness of the service. However, impersonal communication through printed materials and other media forms could discourage clients from exploring clinics and the variety of services that are available. Therefore, it is preferable to communicate with clients by making direct personal contact such as via awareness campaigns and information groups.^25^ Subsequently, efforts to enhance awareness should be concerted and well planned in order to be effective.^19^

Several students reflected that the location is far from their residence and that it is off central campus. A need exists to create awareness and provide specific directions to the service as it is unclear and difficult to locate the clinic. If a facility is not available within a short distance, it is perceived by clients to be out of reach. Upon creating awareness of the service, the geographical location of the healthcare facility should be enhanced.^26^ An efficient social marketing intervention contains a number of key element s such a as consumer orientation, a mutually beneficial exchange and long-term planning. The social marketer seeks to build a relationship with target consumers over time.

The findings indicated that clients are not well informed about CHS and the various services that are rendered. Therefore, a need exists to increase marketing strategies of the facility. Marketing and advertising of the service can be delivered through various sources and channels such as the university’s internal radio broadcasts, campus magazine, the university library and intranet websites.^19^

## Conclusions

It became evident that students and staff members were not aware of the services offered at the CHS, which is a major factor for service attendance. Inadequate marketing and advertising contribute to this factor. Considering the findings that various staff members and students were not knowledgeable about the services rendered, it would be useful for CHS to reconsider their marketing and advertising strategies. Geographical location is an important aspect of healthcare service accessibility. The attendance of clients at the health services decreases as the location distance of the facility increases.^27^ The distribution of facilities not only limits access but also has serious consequences for medical conditions and emergencies.^28^ However, distance alone as a barrier does not fully explain accessibility to facilities.^29^

### Recommendations

Recommendations were made based on the findings of the study. The following recommendations are proposed to improve awareness and address the needs of the campus community. Student and staff members revealed that they have been registered and employed by the university for several years, yet only recently became aware of the service. It is therefore recommended that information regarding the service be advertised in the university newspaper, bill boards, university website and buildings that are often visited by students such as the library. An automated advert could also be created on computers of students and staff.^19^ It would be beneficial for the university community if CHS creates awareness by advertising through sources such as the university radio station, campus magazine and wellness programmes. It is clear that efforts are required to increase awareness, the visibility and the location of the CHS.

A need exists to create awareness and provide specific directions to the service as it is unclear and difficult to locate the clinic. In addition, students who are not residing in the residences close to the CHS should be better informed about the location of the clinic. Further research is recommended to explore the perceptions of clients who access the sexual health clinic on satellite campuses. The distance of healthcare services affects access and attendance of the facility.^29^ It would be beneficial to the university community if the management would consider moving the location of the clinic to a more central geographical location on campus.

### Limitations

The study was conducted at the CHS of SU. Only permanently employed staff members and students enrolled at the main campus who had previously used the service were included in the study. Clients who utilised the service once only were excluded because it was anticipated that they would have limited perceptions regarding the topic under discussion. Sexual health clinics located at the satellite university campuses were not explored because of the geographical distance between main campus and satellite campuses. The first author is employed at the main campus and because of staff shortages was unable to include clients accessing satellite campus clinics because it would affect service delivery at the main campus.
